# Winter climate change mediates the sensitivity of vegetation leaf-out to spring warming in high latitudes in China

**DOI:** 10.3389/fpls.2024.1476576

**Published:** 2024-12-02

**Authors:** Mingyang Chen, Mark Henderson, Binhui Liu, Wanying Zhou, Rong Ma, Weiwei Huang, Zeyu Dou

**Affiliations:** ^1^ College of Forestry, The Northeast Forestry University, Harbin, China; ^2^ Mills College, Northeastern University, Oakland, CA, United States

**Keywords:** vegetation, spring phenology, response efficiency, climate change, minimum temperature, precipitation

## Abstract

Global warming has significantly altered plant phenology by advancing the timing of leaf emergence, impacting vegetation productivity and adaptability. Winter and spring temperatures have commonly been used to explain spring phenology shifts, but we still lack a solid understanding of the effects of interactions between conditions in different seasons. This study utilizes normalized difference vegetation index (NDVI) and meteorological data to examine the effects of changes in winter and spring temperatures and precipitation on the start of the vegetation growing season (SOS) at high latitudes in China from 1982 to 2015. We found that SOS in Northeast China, as a whole, showed a weak advancing trend (moving earlier in the year), but with obvious regional differences. Even within the same vegetation type, changes in SOS were faster in the cold north (1.9 days/decade) and the cold and dry northwest (1.6 days/decade) than the regional averages for deciduous needleleaf forests (DNF; 1.2 days/decade) and grasslands (0.6 days/decade). Increases in spring temperatures dominate forest SOS advancement, while grassland SOS is mainly influenced by winter and spring precipitation. Decreases in winter minimum temperature (T_min_) enhance the spring temperature sensitivity of SOS. The way that winter precipitation regulates the spring temperature sensitivity of SOS differs among vegetation types: increasing sensitivity in grasslands but suppressing it in DNF. The moderating effects of winter conditions account for the greatest part of the regional differences in the magnitude of change in SOS. Our findings highlight that, although rising spring temperatures significantly affect SOS, winter T_min_ and precipitation are crucial for understanding spatial SOS differences, particularly in cold, arid high-latitude regions. Winter conditions play an essential role in regulating the response of vegetation SOS to spring climate at high latitudes. These results suggest that considering the moderating effect of winter climate can facilitate more accurate predictions of temperature-driven phenological changes under future climate change.

## Highlights

- Spring phenology at high latitudes exhibits varying spatial trends.
- Lower winter minimum temperatures enhance the efficiency of vegetation leaf-out in response to spring warming.
- The regulation effect of winter snow differs between grasslands and needleleaf forests.

## 1 Introduction

Vegetation phenology is a natural phenomenon that occurs in an annual cycle where plants are affected by seasonal climate patterns (White et al., 2009). Changes in phenology are among the most direct indicators of vegetation evolution, strongly controlled by climate change (Shen et al., 2018; Vogel, 2022). The start of the vegetation growing season (start of season or SOS) is an important phenology indicator for terrestrial ecosystems in response to changing climates (Caparros-Santiago et al., 2021; Piao et al., 2019). Changes in SOS can influence the terrestrial carbon and water cycles, with a potential impact on the productivity and stabilization of terrestrial ecosystems (Ji et al., 2021; Xu et al., 2020).

Numerous studies have confirmed that global warming has significantly altered vegetation phenology, with SOS gradually advancing in recent years (Gao and Zhao, 2022; Liu et al., 2022). However, although spring leaf phenology as a whole has shown a clear trend of advancement with warming, there are notable differences among species and across regions. The most pronounced changes in vegetation phenology are mainly at high latitudes, and the trend is greater for woody plants than for herbaceous plants (Cheng et al., 2021; Chu et al., 2021; Zhao et al., 2015). In general, spring leaf emergence is co-regulated by a series of factors that vary from environment to environment (Chuine et al., 2016).

In temperate zones, precipitation and, especially, temperature are considered to be the most important environmental factors controlling the phenology of plants (Du et al., 2019a; Zhang et al., 2022a). Warmer temperatures in early spring have been shown to promote earlier vegetation germination (Garzón et al., 2024; Montgomery et al., 2020; Park et al., 2020; Piao et al., 2015), with the highest correlations between spring temperatures and SOS found in the Northern Hemisphere (Chen et al., 2023a; Li et al., 2021). Studies modeling phenological changes based on average daily temperatures, though, ignore the potentially different contributions of daytime and nighttime warming to plant development (Peng et al., 2013; Zhong et al., 2023). Diurnal warming patterns may play different or even opposing roles in spring phenological changes (Meng et al., 2020).

Precipitation also has a very important effect on SOS. Precipitation plays a crucial role in regulating vegetation growth and community structure in many regions (Chen et al., 2023b, 2024; Shen et al., 2015a), especially arid and semi-arid regions (Hu et al., 2022; Korell et al., 2021; Small et al., 2018). Moreover, Shen et al. found that even in swamp ecosystems with favorable moisture conditions, vegetation growth can be limited by changes in precipitation (Shen et al., 2023).

In general, there is still no consensus regarding the linearity of the SOS response to climate change, especially given the fact that shifts in SOS vary spatially and temporally. The sensitivity of phenology to temperature appears to have weakened in recent decades, especially in Europe (Peng et al., 2018; Wang et al., 2019). The mediating effect of winter temperatures on SOS has been proposed as a possible factor (Chamberlain and Wolkovich, 2021; Fu et al., 2015a), but how it does so remains controversial. The existence of complex abiotic–biotic relationships suggests that the ecological memory of winter climate may alter the response of SOS to spring warming (Ogle et al., 2015). Some studies have posited that the accumulated amount of winter cold temperatures affects the amount of forcing needed to trigger germination bursts and thus affects SOS (Ettinger et al., 2020). Germination of plant leaves in temperate and boreal regions usually requires a certain cold temperature accumulation to break bud endodormancy, followed by the arrival of warmer conditions (known as forcing temperatures) to trigger cell growth and leaf development (Man et al., 2017). However, other researchers have proposed that it is the number of cold winter days, rather than the accumulated cold temperatures, that is the main factor affecting SOS (Beil et al., 2021). Winter precipitation also affects spring phenology at mid- to high latitudes in the Northern Hemisphere in a complex way (Jin et al., 2019; Yun et al., 2018). The SOS may be advanced or delayed by a few days or even weeks depending on changes in precipitation during the pre-season period (Li et al., 2023; Wang et al., 2022a).

Characterizing how winter climate mediates the response of vegetation SOS to warming spring temperatures is critical to bridging this conceptual disconnect and better understanding how SOS is likely to change as the climate warms. However, studies addressing this issue to date have focused on average winter temperatures and have been mainly conducted on single species through controlled experiments and *in situ* observational studies rather than (Man et al., 2017; Zhang et al., 2022b, 2018) comparing different vegetation types at large spatial scales. In addition, little consideration has been given to potential differences in the effects of daytime and nighttime temperatures or to the possible effects of changes in winter precipitation. These gaps limit our ability to predict regional and global changes in SOS in a warming climate and the resulting changes in ecosystem structure and function.

The methods of phenological observation mainly include ground observation and remote sensing (Berra and Gaulton, 2021; Gao and Zhang, 2021). Ground observations are traditionally carried out by directly monitoring plants and climate variables, which may limit the study to a small area and a few species. Remote sensing monitoring compensates for the shortcomings of ground-based observation, providing a data source for community-scale and regional-scale vegetation phenology studies and becoming a widely used tool for phenology research (Smith and Ramsay, 2020; Wang et al., 2022b), although it too is limited to the temporal, spatial, and spectral resolution of the remote sensor data (Gong et al., 2024; Piao et al., 2019).

In this paper, we used GIMMS 3g normalized difference vegetation index (NDVI) and meteorological data from 1982 to 2015 to investigate the spatial and temporal characteristics of SOS of different vegetation types and their responses to winter and spring climate change. Our study focuses on Northeast China, which is located in the mid- to high latitudes of the Northern Hemisphere and is highly sensitive to climate change, mainly focusing on the moderating role of winter climatic conditions on the efficiency of vegetation SOS in response to spring warming. We make the following assumptions: 1) winter precipitation’s role has a certain connection with vegetation type, and 2) there is a diurnal difference in winter temperatures’ regulation effect. Our aim was to enhance the ability to understand and predict changes in vegetation phenology under warming conditions. Our results may provide valuable insights for predicting future changes in SOS, as well as in vegetation productivity and adaptability, on which to base forest management decisions to cope with global warming.

## 2 Materials and methods

### 2.1 Study area

**Figure 1:**
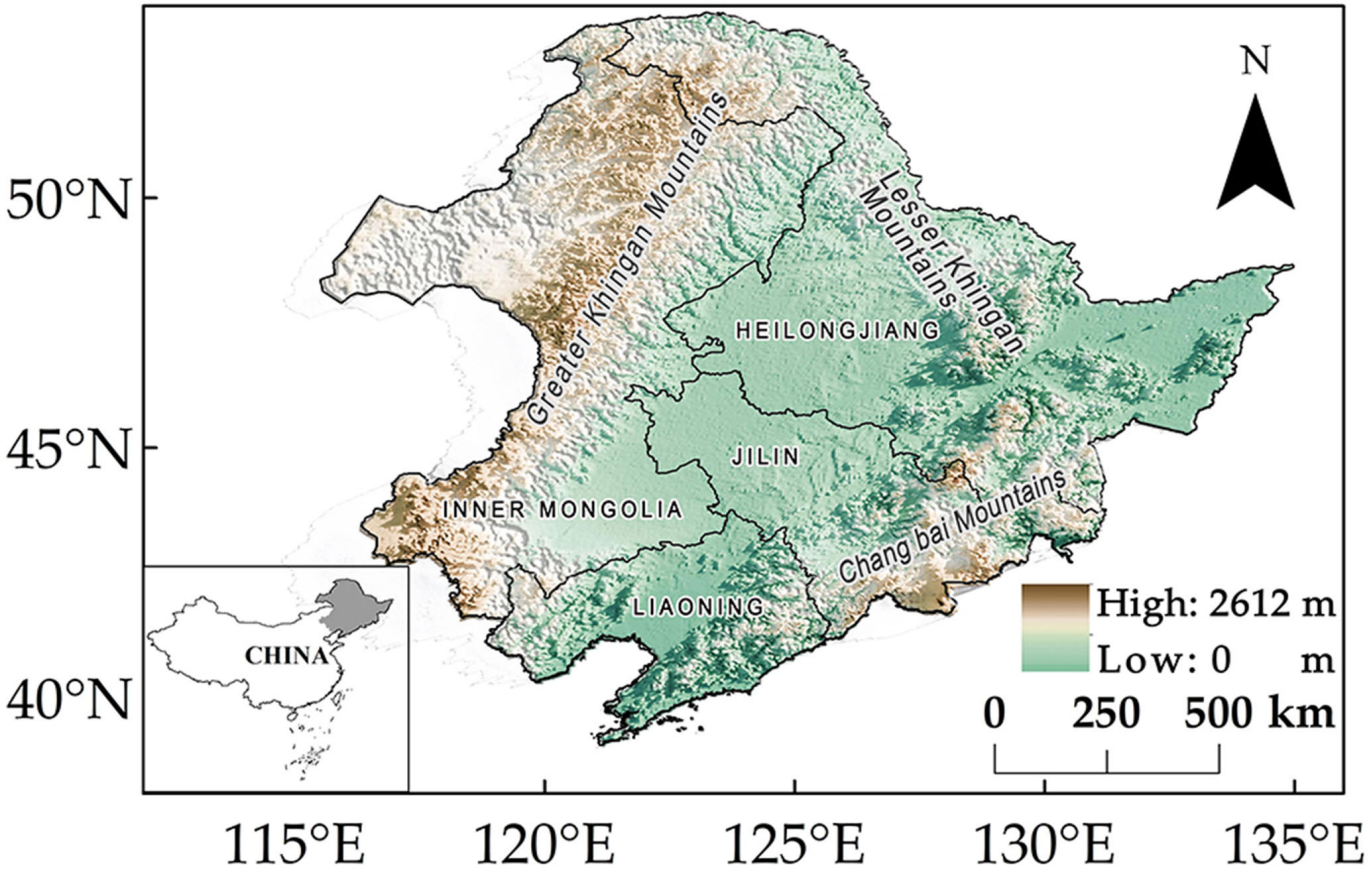
Location of study area and its elevation.

The study area belongs to the middle and high latitudes of the Northern Hemisphere and is located in Northeast China (**Figure 1**) (38°72′–53°55′N, 115°52′–135°09′E), which is known for high sensitivity to global climate change. It administratively comprises the provinces of Heilongjiang, Jilin, and Liaoning, as well as three cities and one league (Tongliao, Chifeng, Xing’anmeng, and Hulunbei’er) of eastern Inner Mongolia (Zhang et al., 2023). The study area encompasses a total of approximately 1.27 million km^2^. This region, most of which belongs to the temperate continental monsoon climate zone, is one of the largest forest carbon pools in China (Qiu et al., 2020; Sun and Liu, 2019).

**Figure 2:**
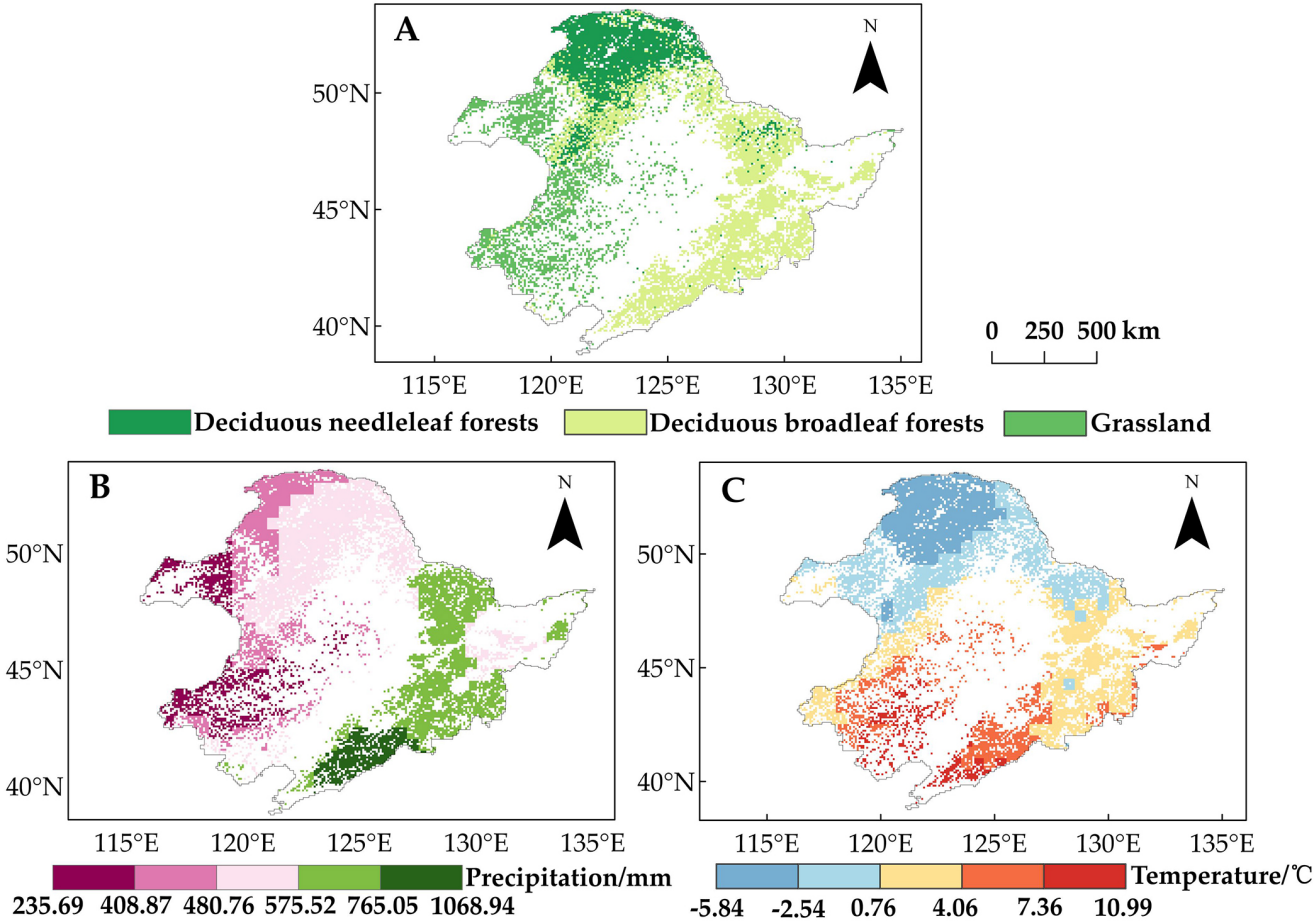
**(A)** Distribution of the three vegetation types. **(B)** Spatial distribution of average annual precipitation in Northeast China from 1982 to 2015. **(C)** Spatial distribution of average temperature in Northeast China from 1982 to 2015.

The study area encompasses a wide range of spatial variability in climate conditions, characterized by a longitudinal gradient in precipitation (higher in the east and lower in the west) and a latitudinal gradient in temperature (higher in the south and lower in the north), with an east–west difference of 833 mm in annual average precipitation (**Figure 2B**) and a north–south difference of 16.83°C in annual average temperature (**Figure 2C**). The region’s diverse vegetation types (**Figure 2A**) (Luo et al., 2020) allow for a comparative study of the effects of a warming climate on vegetation phenology.

### 2.2 Data

Satellite-derived NDVI data covering the 1982–2015 period were obtained from the GIMMS NDVI 3g dataset, which has a spatial resolution of 8 km × 8 km and a temporal resolution of 15d. This global vegetation index change dataset was produced by the U.S. National Aeronautics and Space Administration (NASA) (http://ecocast.arc.nasa.gov/data/pub/gimms/3g.v1) and is appropriate for determining long-term trends in global vegetation activity.

Land use data were accessed from the European Space Agency (ESA) Climate Change Initiative (CCI) annual land cover dataset (https://maps.elie.ucl.ac.be/CCI/viewer/download.php), which has a spatial resolution of 300 m and an estimated classification accuracy of more than 70%. Areas with no change in vegetation cover from 1982 to 2015 were selected to categorize the land cover types in the study area, including deciduous needleleaf forests (DNF), deciduous broadleaf forest (DBF), and grassland (**Figure 2A**).

We accessed meteorological data from the CRUTS v4.07 dataset produced by the UK National Centre for Atmospheric Science (NCAS) (https://crudata.uea.ac.uk/cru/data/hrg), which has a spatial resolution of 0.5° and spans the time period 1901–2022. We extracted the 1982–2015 period monthly maximum temperature (T_max_), minimum temperature (T_min_), and precipitation values and calculated the seasonal averages for each year, i.e., winter (December from the previous year to February of the current year) and spring (“March to May” of the current year). We resampled the land use data and meteorological data to be consistent with the 8-km spatial resolution NDVI data (Deng et al., 2019; Zhang et al., 2022c).

### 2.3 Methods

#### 2.3.1 Extraction of vegetation phenology

In this study, the Polyfit-Maximum method was used to determine SOS. This method is based on the principle that the first period of significant increase in the vegetation index (NDVI) each year marks the SOS (Piao et al., 2006). Compared to other methods for extracting candidate SOS dates, this method reduces the effects of aerosols, clouds, and other factors (Jeong et al., 2011). Numerous studies have been conducted using this method to calculate the SOS (Ma et al., 2022; Shen et al., 2019; Yuan et al., 2020). Following this method, the multi-year average of NDVI was first calculated to derive the NDVI time series with an interval of 15 days. Then, the temporal rate of change of NDVI was calculated according to Equation 1:


(1) $$NDVIratio\;\left(\text{t}\right)\;=\frac{NDVI\left(\text{t}+1\right)-NDVI\left(\text{t}\right)}{NDVI\left(\text{t}\right)}\;\;\;\;\;\;\;$$

where the NDVIratio(*t*) is the rate of change of NDVI at time *t*, *t* is a certain moment (calculated from January 1), and (*t* + 1) indicates the next moment, with an interval of 15 days.

NDVI as computed from satellite remote sensing imagery often has some outliers due to non-vegetation effects such as clouds, atmospheric interference, and the solar radiation angle. In order to minimize the influence of the outliers on the phenological assessments, the polynomial maximum method was used, and the sixth-degree polynomial was found to better fit the NDVI time series (Equation 2):


(2) $$NDVI\left(t\right)\;=\;a\;+\;a_{1}t+\;a_{2}t^{2}+\;a_{3}t^{3}+\;a_{4}t^{4}+\;a_{5}t^{5}+\;a_{6}t^{6}$$

where NDVI(*t*) is the threshold value corresponding to day *t* calculated in Equation 1 and *a*1, *a*2, *a*3, …, *a*6 are the fitting parameters, which are calculated by least squares regression.

#### 2.3.2 Trend analysis

We used the one-dimensional linear regression method (Shen et al., 2019) to analyze the trend of SOS and climate indicators at the regional scale and the image element scale. The trend was calculated as shown in Equation 3:


(3) $$\text{Slope}=\frac{n*\sum_{i=1}^{n}\;i\;*\;P_{i}\;-\;\left(\sum_{i=1}^{n}i\;\right)\left(\sum_{i=1}^{n}P_{i}\;\right)}{n*\sum_{i=1}^{n}i^{2}\;\;-\;\left(\sum_{i=1}^{n}i\right)2}$$

where Slope indicates the slope of the regression equation for variable *P*, *Pi* is the value of the variable in year *i*, and *n* is the number of years in the study period. When the slope is positive, it indicates that a delay in SOS has occurred; conversely, a negative slope signals that SOS is advancing (occurring earlier in the year).


(4) $$E_{sos}=\left(R_{sos}-T_{sos}\right)/T_{sos}$$

where R*_sos_* indicates the average SOS trend for each of the four special study regions, T*_sos_* indicates the average SOS trend for the vegetation type corresponding to each region, and E*_sos_* indicates the rate of increase or decrease in R*_sos_* compared to T*_sos_*.

#### 2.3.3 Partial correlation analysis

To exclude the interference of other variables and explore the relationships between individual climate indicators and SOS separately, we used a partial correlation analysis to analyze the relationship between seasonal climate indicators and SOS for each period. The formula is shown in Equation 5 (Su et al., 2022):


(5) $$r_{xy.z}=\frac{r_{xy}\;-\;r_{xz}r_{yz}}{\sqrt{\left(1-r_{xy}^{2}\right)\left(1-r_{yz}^{2}\right)}}$$

where r_xy.z_ is the partial correlation coefficient between *x* and *y* after the variable *z* is kept fixed, r_xy_ is the relationship between *x* and *y*, r_yz_ is the correlation coefficient between *y* and *z*, and r_xz_ is the difference between the *x* and *z*.

The significance of the partial correlation coefficients in this paper was examined using t-tests. To further analyze the effect of climate change on SOS, this paper also compared the trends of SOS and the partial correlation coefficients of SOS with T_max_ and T_min_ under different temperature and precipitation trends in winter (Shen et al., 2023).

## 3 Results

### 3.1 Spatial and temporal variations in the SOS

**Figure 3:**
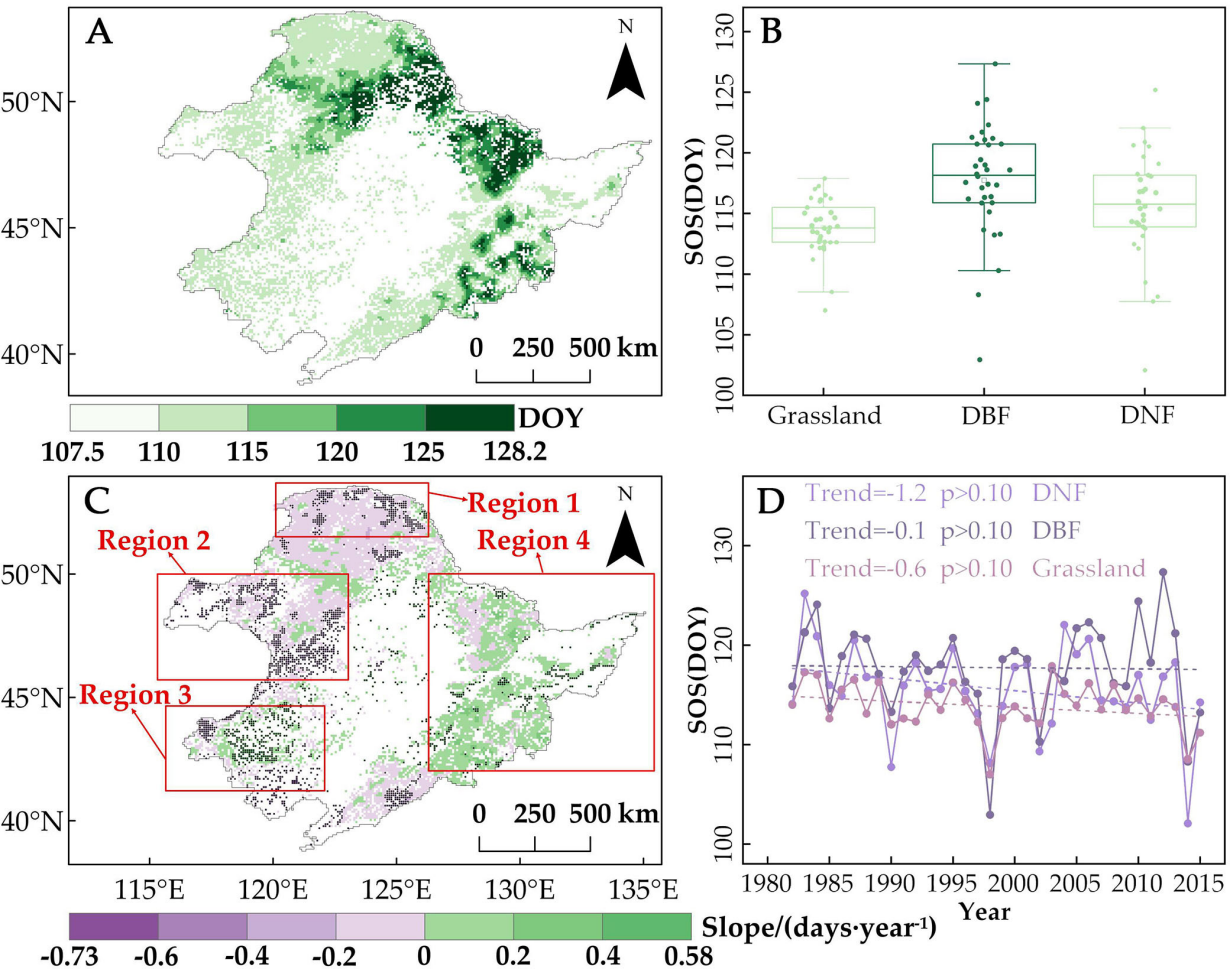
**(A)** Spatial distribution of multi-year average of the start of the vegetation growing season (SOS) by day of the year (DOY) in Northeast China, 1982–2015. **(B)** Box plot of multi-year average SOS values for grasslands, deciduous needleleaf forests (DNF), and deciduous broadleaf forests (DBF). Lighter to darker colors correspond to panel A representing earlier to later SOS, on average. The box represents the middle 50% of the SOS data, the line inside the box represents the median, the vertical lines extend to the minimum and maximum values of the dataset, and the data points that lie outside the maximum and minimum values are the extreme values. **(C)** Spatial trends in SOS from 1982 to 2015. Pixel points with dots indicate significant trends (*p* < 0.05). Red boxes mark the subregions focused on in this study. **(D)** Trends in regional average SOS (days per decade) from 1982 to 2015.

The spatial distribution of the vegetation SOS in Northeast China from 1982 to 2015 is shown in **Figure 3A**. The average SOS ranged from 107.5 to 128.2 the day of the year (DOY), from mid-April to early May, with an average SOS of 115.7 DOY for the entire study area. SOS dates differ among the different vegetation types and different distribution areas of the same vegetation type. The average SOS of grassland was the earliest (113.8 DOY), followed by DNF (115.6 DOY) and then DBF (117.7 DOY). By location, DBF SOS appeared earlier in the southern and lower elevation portions of the study area and later in the more northern, higher elevation areas of the Changbai Mountains, Lesser Khingan Mountains, and eastern Greater Khingan Mountains.

From 1982 to 2015, on average, DNF advanced by 1.2 days per decade, DBF by 0.1, and grassland by 0.6 (**Figure 3D**). However, the trend of SOS showed obvious spatial differences, with some pixels showing significant changes. Spatially, the pixels showing an advancing trend in SOS of DNF accounted for 81.9% (12.2%, *p* < 0.05), mainly focused on the northern region of the Greater Khingan Mountains (**Figure 3C**). The advancing trend in SOS was especially pronounced along the northern boundary of the study area (**Figure 3C**, Region 1), with a significant trend averaging 1.9 days per decade. This represents a 58% faster rate of advancement compared with the regional average trend for DNF SOS.

The proportion of grassland pixels showing an advancing trend in SOS was 62.9% (31.4%, *p* < 0.05), with the area with significant advancement trends concentrated in the northwestern and central portions of the study area (**Figure 3C**, Region 2). On average, Region 2 grassland SOS advanced by 1.6 days/decade, which is 167% faster than the regional average trend for grasslands (**Figure 3D**). However, the southwestern part of the study area, which is mainly grassland, saw a significant delaying trend of 2.6 days/decade (**Figure 3C**, Region 3). The majority of the DBF pixels located in the eastern part of the study area saw a delay in SOS (60.3%, including 6.1%, *p* < 0.05) (**Figure 3C**, Region 4), with an average delay of 1.2 days/decade, while the proportion of pixels showing an advancing trend was only 38.8% (7.6%, *p* < 0.05), mainly focused on the southern part of the Changbai Mountains.

**Figure 4:**
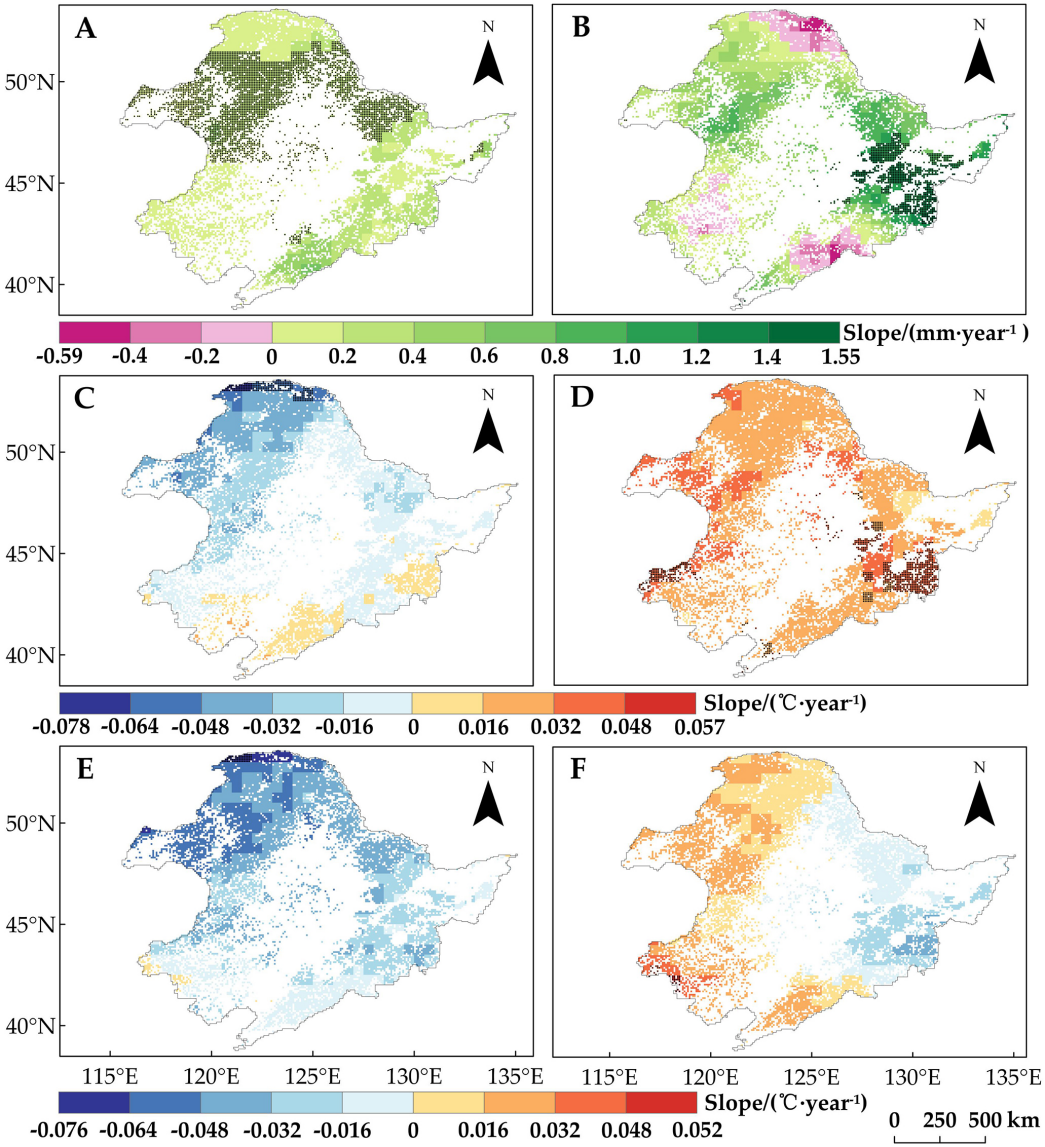
Trends of regional climate indicators in Northeast China from 1982 to 2015. **(A)** Winter precipitation. **(B)** Spring precipitation. **(C)** Winter T_min_. **(D)** Spring T_min_. **(E)** Winter T_max_. **(F)** Spring T_max_. Pixel points with dots indicate significant trends (*p* < 0.05).

In order to explain the spatial variability of SOS trends in the study area across different regions and vegetation types, we analyzed the changes in winter and spring precipitation and temperature during the period of 1982–2015 (**Figure 4**). We found that winter precipitation (**Figure 4A**) and spring T_min_ (**Figure 4D**) generally showed an increasing trend across the study area. In contrast, changes in spring precipitation (**Figure 4B**), spring T_max_ (**Figure 4F**), and winter temperature (T_max_ and T_min_) (**Figures 4C, E**) exhibited significant spatial variations. In particular, winter temperature (T_min_ and T_max_) showed a decreasing trend north of the Greater Khingan Mountains and in the northwestern and central parts of the study area, especially in Region 1 and Region 2, where the SOS significantly advanced. Spatial variability in winter temperatures may account for some of the spatial differences in vegetation SOS change, but the spatial differences in spring T_max_ and precipitation change may also contribute. Therefore, to further determine the reasons for the spatial variation in SOS change, we analyzed the relationship between SOS and seasonal climate change.

### 3.2 Relationships between the SOS and climate indicators

**Figure 5:**
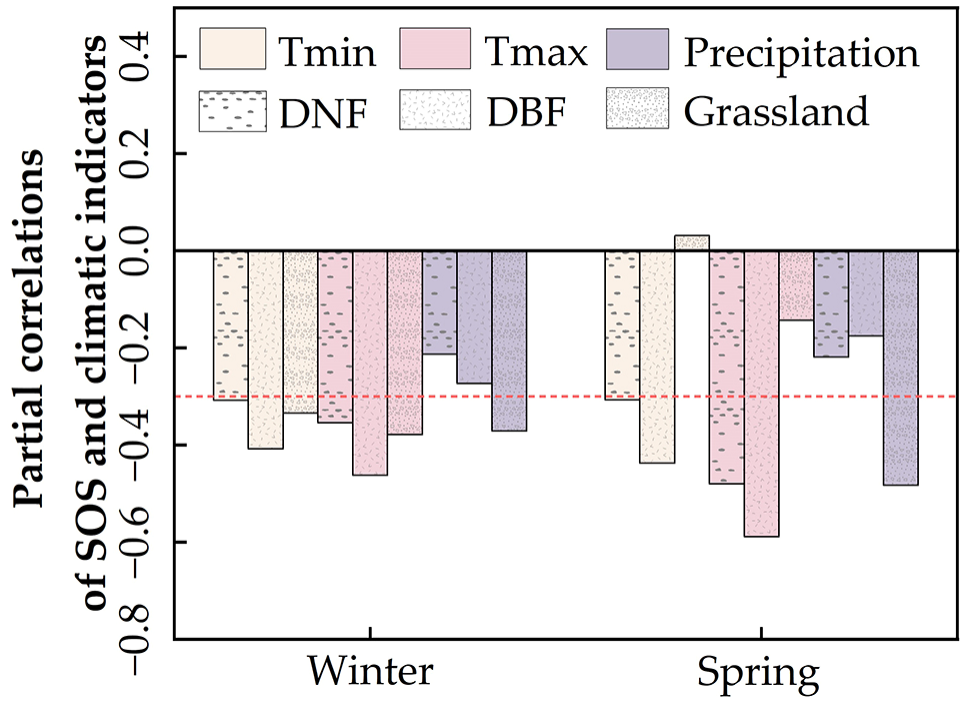
Partial correlations between regional average start of the vegetation growing season (SOS) and climate indicators.

We first analyzed the partial correlation between average SOS and climate indicators for different vegetation types (**Figure 5**) and found that the SOS of forests (especially DBF) was strongly influenced by temperature. Forest SOS showed significant negative correlations with both winter and spring temperatures (T_min_ and T_max_) and a weaker negative correlation with precipitation. Grassland SOS was co-regulated by T_min_, T_max_, and precipitation, showing significant negative correlations with winter temperatures (T_min_ and T_max_) as well as winter and spring precipitation while showing weaker correlations with spring temperatures.

**Figure 6:**
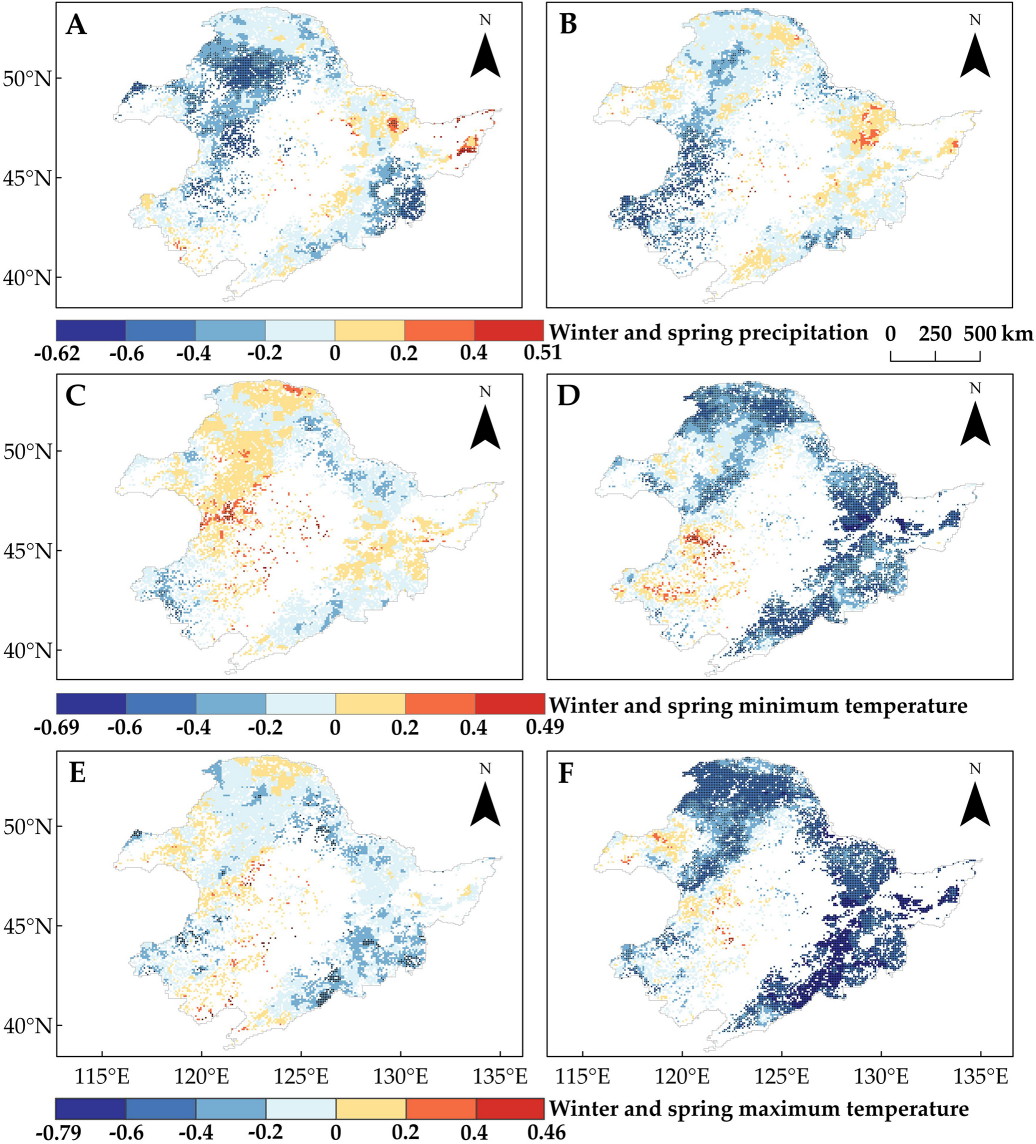
Partial correlation analysis of the start of the vegetation growing season (SOS) with climate indicators from 1982 to 2015. Pixel points with dots indicate significant trends (*p* < 0.05). **(A)** Winter precipitation. **(B)** Spring precipitation. **(C)** Winter T_min_. **(D)** Spring T_min_. **(E)** Winter T_max_. **(F)** Spring T_max_.

On a pixel-by-pixel basis (**Supplementary Tables S1**, **S2**), DNF SOS and DBF SOS were most affected by spring T_max_, followed by spring T_min_; the proportions of pixels with negative correlations between SOS and spring T_max_ were 100% (85.26%, *p* < 0.05) for DNF and 99.85% (86.9%, *p* < 0.05) for DBF (**Figure 6F**); for spring T_min_, they were 99.42% (52.99%, *p* < 0.05) and 99.13% (65.24%, *p* < 0.05), respectively (**Figure 6D**). This is the same as the partial correlation between average SOS and climate factors (**Figure 5**).

While the effect of precipitation on grassland SOS was similar, that relationship showed obvious spatial differences across seasons: the pixels with significant negative correlations between grassland SOS and winter precipitation (27.68%, *p* < 0.05) were mainly concentrated in the northwestern and central (Region 2) parts of the study area (**Figure 6A**), while for spring precipitation (35.84%, *p* < 0.05), they were mainly concentrated in the southwest (Region 3) (**Figure 6B**). In terms of the effect of precipitation on forest SOS, unlike the weak correlation with regional average SOS (**Figure 5**), there were regions of significant negative correlations between winter or spring precipitation and SOS for both DNF (26.74% and 3.6%, *p* < 0.05) and DBF (18.55% and 39.78%, *p* < 0.05) (**Figures 6A, B**).

Although winter temperatures were negatively correlated with the trends of grassland and DNF SOS overall (**Figure 5**), we found a positive partial correlation in portions of the study area, particularly in the northwestern and central parts (Region 2) and the northern boundary (Region 1) (**Figures 6C, E**). Winter T_min_, in particular, showed a positive correlation for approximately half of the study area pixels (54.88% for DNF and 47.03% for grassland). This was true for smaller portions of the grassland area: spring T_min_ (15.8%, *p* < 0.05) and T_max_ (6.55%, *p* < 0.05) (**Figures 6D, F**). The partial correlations between SOS and climate indicators indicate that the climate response of SOS differs among regions of Northeast China, even for the same vegetation type (**Figure 6**).

**Figure 7:**
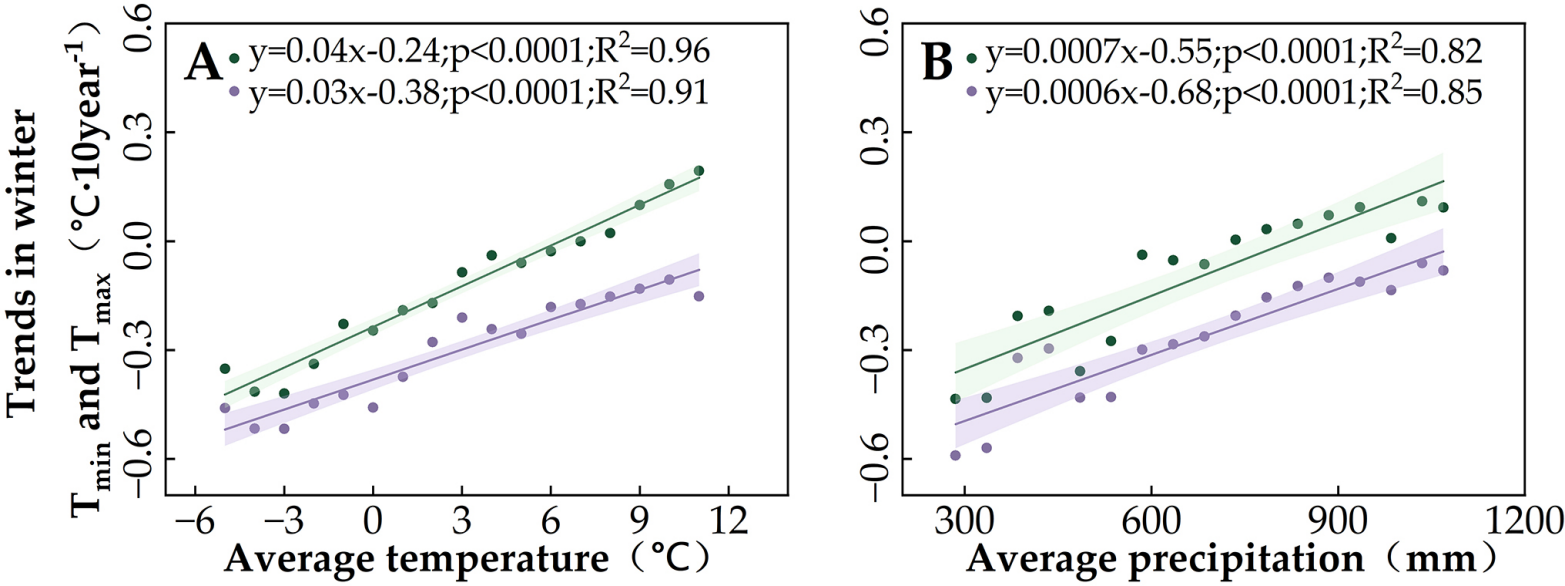
Influence of multi-year average temperature and precipitation conditions on winter T_min_ and T_max_ trends in the study area: **(A)** average temperature and **(B)** average total precipitation. Purple indicates T_max_, and green indicates T_min_. The lines indicate the linear fit for the winter temperature trends, and the shading represents the 95% confidence band.

Spatial variability in the partial correlations between SOS and these climate indicators may explain the differences in SOS changes (**Figure 3C**). We found that the regions where winter temperatures showed positive effects on SOS overlapped with the regions where SOS significantly advanced (Regions 1 and 2). In light of winter temperature trends (**Figures 4C, E**), we noted that the decrease in T_min_ and T_max_ in winter promotes the advancement of SOS. This phenomenon is more pronounced in Regions 1 and 2, which are located in cold and arid regions (**Figure 7**). Moreover, by comparing the positive correlations between SOS and winter T_min_ or T_max_ (**Figures 6C, E**) in the context of the SOS trends (**Figure 3C**), we found that winter T_min_ is the most important driver of changes in vegetation SOS.

**Figure 8:**
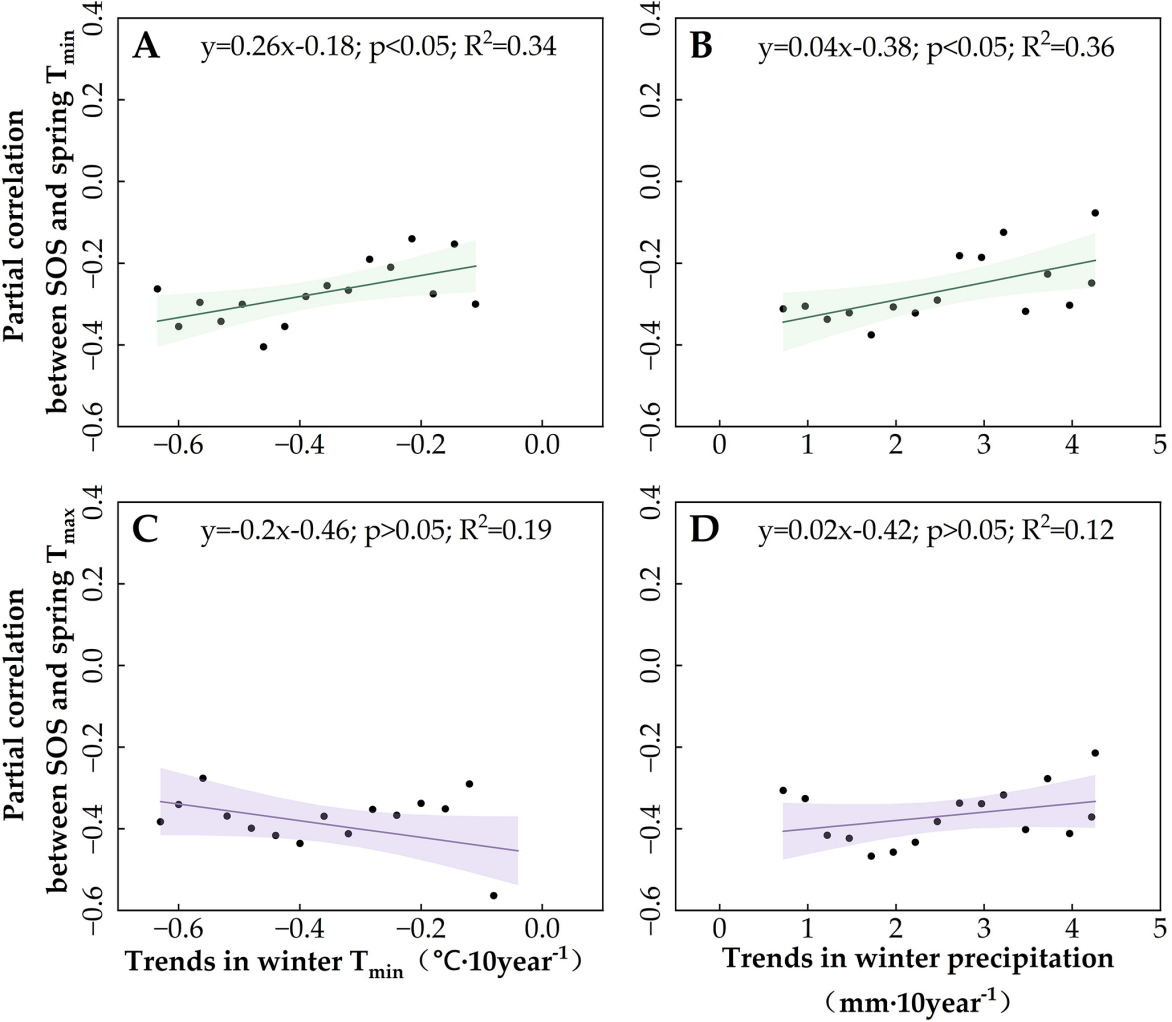
Moderating role of winter climate indicators in Region 1 [deciduous needleleaf forests (DNF)] on the spring temperature sensitivity of the start of the vegetation growing season (SOS). **(A)** The effect of winter T_min_ trends on the partial correlation between SOS and T_min_. **(B)** The effect of winter precipitation trends on the partial correlation between SOS and T_min_. **(C)** The effect of winter T_min_ trends on the partial correlation between SOS and T_max_. **(D)** The effect of winter precipitation trends on the partial correlation between SOS and T_max_. The lines indicate the linear fit for the partial correlation coefficient, and the shading represents the 95% confidence band.

**Figure 9:**
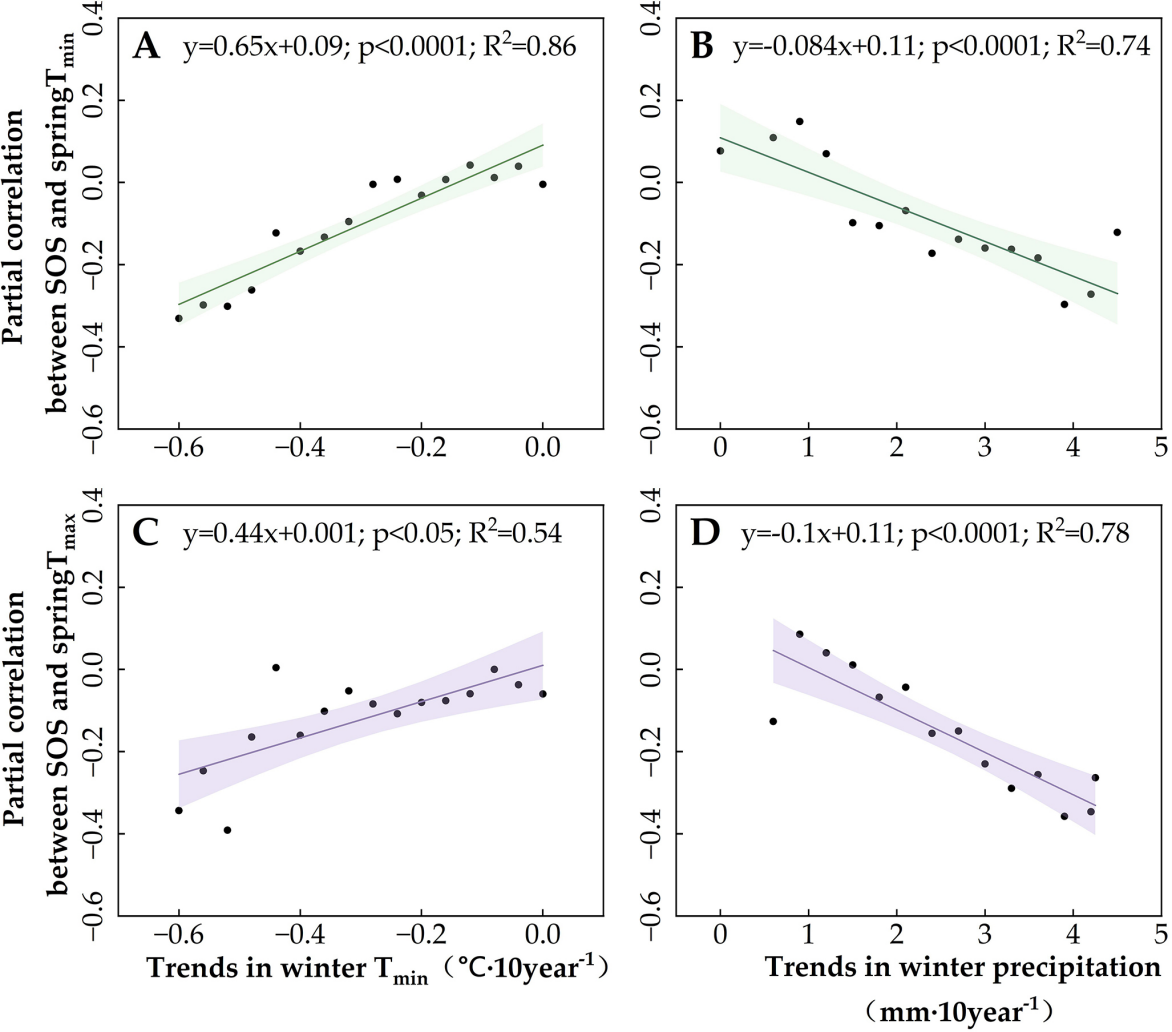
Moderating role of winter climate indicators in Region 2 (grassland) on the spring temperature sensitivity of the start of the vegetation growing season (SOS). **(A)** The effect of winter T_min_ trends on the partial correlation between SOS and T_min_. **(B)** The effect of winter precipitation trends on the partial correlation between SOS and T_min_. **(C)** The effect of winter T_min_ trends on the partial correlation between SOS and T_max_. **(D)** The effect of winter precipitation trends on the partial correlation between SOS and T_max_. The lines indicate the linear fit for the partial correlation coefficient, and the shading represents the 95% confidence band.

Subsequently, we examined the effects of winter temperature decreases on the SOS trend (**Supplementary Figures S1A**, **S2A**), as well as the partial correlation of SOS with spring temperatures in Region 1 (primarily DNF) and Region 2 (primarily grassland) (**Figures 8**, **9**) so as to clarify the moderating role of winter temperatures on SOS and its response to spring temperatures. The results showed that the advancing trend of SOS (**Supplementary Figures S1** and **S2**) and the negative partial correlation between spring T_min_ and SOS increased gradually with the magnitude of the winter T_min_ decline (**Figures 8A**, **9A**). However, the effect of winter T_min_ on the partial correlation between SOS and spring T_max_ differed between Regions 1 and 2. Greater declines in winter T_min_ increased the partial correlation between SOS and spring T_max_ (**Figure 8C**) in the grasslands of Region 2 but had no effect on the DNF in Region 1 (**Figure 9C**). This suggests that a decrease in winter T_min_ can increase the rate of the SOS response to spring temperatures, but the regulatory effect differed between the vegetation types. For DNF, this moderating effect was focused on the response of SOS to spring T_min_, whereas for grasslands, the response of SOS to both spring T_min_ and T_max_ was modulated by winter T_min_.

Because SOS in both Region 1 DNF and Region 2 grassland are also affected by winter precipitation (**Figure 6**), we also analyzed the effect of increases in winter precipitation on the trend of SOS (**Supplementary Figures S1B**, **S2B**) and the partial correlation of SOS with spring temperatures (**Figures 8B, D**, **9B, D**). We found that changes in winter precipitation trends had completely opposite effects on SOS in Region 1 and Region 2. For DNF in Region 1, we found that increased winter precipitation is associated with slower advancement of SOS (**Supplementary Figure S1B**) and a reduction in the partial correlation between SOS and spring T_min_ (**Figure 8B**), but not spring T_max_ (**Figure 8D**). By contrast, for grasslands, winter precipitation has the same effect as winter T_min_: both are associated with a faster-advancing trend in SOS and greater partial correlations of SOS with spring T_min_ and T_max_. Thus, the changes in thermal and moisture conditions in the wintertime, not only spring warming, are of primary importance for vegetation SOS. The effects of winter and spring conditions on SOS are likely to be different, with winter climate change influencing the speed of the effect of spring warming.

In Region 3 (primarily grasslands) and Region 4 (primarily DBF), the effect of changes in winter T_min_ and precipitation was weaker than that in Regions 1 and 2. For Region 3, neither changes in winter T_min_ nor precipitation had an obvious effect on grassland SOS (**Supplementary Figure S3**). For Region 4, the negative correlation of SOS with spring T_min_ and T_max_ increases slightly with an increase in the magnitude of winter T_min_ and precipitation trend (**Supplementary Figures S4C, E, F**). This contrasts with our finding in Region 1 that DNF SOS sensitivity is regulated by winter T_min_ (**Supplementary Figure S1C**).

## 4 Discussion

### 4.1 Characterization of SOS response to climate change

From 1982 to 2015, the overall advancement in average SOS in the study area was consistent with the trend of early greening of the vegetation in the Northern Hemisphere at middle and high latitudes (Jeong et al., 2011; Jiang et al., 2023; Schwartz et al., 2006). The average SOS ranged from 107.5 to 128.2 DOY with an average SOS of 115.7 DOY for the entire study area. This is similar to previous studies showing SOS dates of 100–130 DOY in Northeast China (Qiao et al., 2019; Zhang et al., 2022c). The average SOS of grassland was the earliest (113.8 DOY), followed by that of DNF (115.6 DOY) and then DBF (117.7 DOY), which can be attributed to the fact that the woody plants are less sensitive to the climate than herbaceous plants (Post and Stenseth, 1999). For woody plants, the different water transport structures of conifers and broadleaf trees affect the water supply in the face of low late-winter temperatures, while conifers are virtually unaffected, which may contribute to the fact that the DNF SOS precedes the DBF SOS (Cochard and Tyree, 1990; Wang et al., 1992).

Since the SOS in the study area was focused on mid-April to early May, changes in winter and spring climates were taken into account in analyzing the effect of climate on SOS. There were differences in the response of regional average SOS to climatic change among the different vegetation types, with forest SOS being mainly influenced by spring T_min_ and T_max_ and weakly influenced by precipitation, whereas grassland SOS is more affected by winter and spring precipitation. Our results are consistent with the results of previous studies (Hernández Ayala et al., 2021; Wang et al., 2022c; Zhao et al., 2024). Previous studies have confirmed the important role of precipitation and temperature on vegetation growth (Chen et al., 2018; Jiao et al., 2021; Zhang et al., 2022a).

However, unlike the opposite response of vegetation growth to diurnal warming reported by Meng et al. (2020), we found that forest SOS was significantly and negatively correlated with both spring T_min_ and T_max_, although the correlation is higher with spring T_max_. This is because the growth of vegetation is controlled by the balance of photosynthesis (in the daytime) and respiration (at night) (Peng et al., 2013; Sun et al., 2023). SOS, though, is not simply a function of ordinary vegetation growth: our study focuses on the process of new leaves appearing in spring. The process of growing new leaves relies upon the preceding year’s stored organic matter, and when plants break out of winter dormancy to grow new leaves, they depend mainly on spring temperature and moisture conditions (Jiao et al., 2021; Zhang et al., 2022a). The increase in spring T_min_ and T_max_ may accelerate heat accumulation that favors early SOS (Zhang et al., 2023).

In response to winter T_min_ and T_max_, the regional average SOS of all three vegetation types showed consistent and significant negative correlations, with the most significant correlation in DBF. This can be explained by spatial variation in the role of winter temperatures, with SOS responding positively to winter T_min_ in some areas of DNF and grassland, weakening the overall negative correlation between regional average SOS and winter T_min_. Our results show that an increase in spring T_max_ and spring T_min_ can meet the heat accumulation threshold required for vegetation growth and significantly promote the advancement of SOS in forests, similar to previous findings on temperate woody plants (Fu et al., 2015b; Zhang et al., 2023). Grasslands, in contrast, are more sensitive to precipitation because they are located in drier regions. In addition, since herbaceous plants have shallower root systems and are more susceptible to soil moisture fluctuations than forests (Chen et al., 2015), changes in spring and winter precipitation significantly affect the SOS of grassland.

### 4.2 The moderating role of winter climate on vegetation SOS sensitivity to spring climate

This study examined in detail the characteristics of SOS changes in four regions of Northeast China, finding significant trends in Regions 1–3. When we attributed the SOS changes to climate indicators, we found that this study area has experienced a trend of lower winter temperatures (T_min_ and T_max_)—unlike the general trend of global warming (Li et al., 2015a; Luo et al., 2024; Richardson, 2022; Scafetta, 2024) and winter temperatures in most of the world (McCabe and Wolock, 2010). This is mainly due to the fact that our study period of 1982–2015 includes the so-called “warming hiatus” of 1998–2012, during which winter cooling was experienced in some terrestrial biomes, temporarily obscuring the continued rise in global temperatures (Li et al., 2015b, 2015; Trenberth et al., 2014). Other studies have also noted the occurrence of decreasing winter T_min_ and T_max_ in high-latitude boreal forests around this time (Cohen et al., 2012; Deng et al., 2019).

The study area overall showed increases in spring T_min_ and T_max_ but a decrease in winter T_min_, resulting in a significant advancing trend of DNF SOS. In most of Region 1, the northernmost and coldest region, dominated by DNF, the SOS showed a significant advancing trend that overlapped with the area where winter T_min_ was positively correlated with SOS. The partial correlation of DNF SOS with winter T_min_ here was positive, and the partial correlations with spring T_min_ and winter precipitation were significantly negative.

As previously noted, SOS in most cold-temperate plants is the result of the interaction between winter cold and spring heat (Linkosalo et al., 2008; Luedeling et al., 2009; Park et al., 2018). Temperate vegetation overwinters in a dormant state to protect sensitive growing tissues from frost damage; coming out of dormancy and resuming growth in the spring involve meeting a required threshold of winter cold and then accumulating spring heat (Arlo Richardson et al., 1974; Luedeling et al., 2009). Previous studies on the effects of winter and spring temperature changes on SOS have focused on the effects of average temperatures on vegetation (Yu et al., 2010; Zhang et al., 2022b), which may have led to an underestimation of the effects of cold winter nighttime temperatures on SOS.

Our study found that the role of winter T_min_ was stronger than that of winter T_max_ in the regulation of the spring temperature response of grassland and DNF SOS in cold, arid regions. This is due to seasonal differences in the effects of diurnal temperature on vegetation activity, with SOS mainly affected by T_max_ in spring and by T_min_ in winter (Deng et al., 2019). Nighttime cooling in winter increases the efficiency of spring T_min_ warming on leaf emergence due to a higher accumulation of chilling units; this induces a positive correlation between winter T_min_ and forest SOS (Liu et al., 2024; Meng et al., 2020; Zhang et al., 2022c). While the significant effect of spring T_max_ on SOS makes it unregulated by winter T_min_, this supports our conclusion: it indicates that the effects of winter temperatures on the spring temperature sensitivity of SOS are mainly driven by changes in winter T_min_ and primarily regulate the effect of spring T_min_ on DNF SOS.

In this study, precipitation showed a countervailing effect on DNF SOS: the increasing trend of winter precipitation weakened the advancing trend of SOS in DNF. This is similar to the inhibitory effect of precipitation on the growth of temperate DNF (Fang et al., 2005). Lower winter T_min_ would seem to have met the cold demand threshold for vegetation to break out of dormancy with the arrival of spring warming. However, higher soil temperatures due to increased winter snowpack (Maurer and Bowling, 2014; Sanmiguel-Vallelado et al., 2021; Wilson et al., 2020) instead delayed the fulfillment of the cold demand of vegetation, leading to a weaker trend in the advancement of SOS. Thus, an acceleration in the decreasing trend of winter T_min_ favors an increase in the partial correlation between spring T_min_ and DNF SOS, whereas an uptick in the increasing trend of winter precipitation has an inhibitory effect on the increase in the partial correlation between spring T_min_ and DNF SOS.

In the cold, arid grasslands of Region 2, located in the northwestern and central portions of the study area, the response of grassland SOS to winter T_min_ and spring T_min_ was the same as that of DNF SOS in Region 1. An increase in the downward trend in winter T_min_ in this region had a similar effect, increasing the negative partial correlation of SOS with spring T_min_. In addition, an increase in the magnitude of the decreasing trend of winter T_min_ can also increase the response of SOS to warming spring T_max_. This is because the mechanism by which winter temperature modulates the efficiency of the spring temperature response of grassland SOS is different from DNF SOS. The combination of decreased winter T_min_ and increased winter precipitation favors snow accumulation (Barrere et al., 2018; Zhang et al., 2019). It can provide water available for the following year’s plant growth as spring temperatures rise, reducing the demand for plants for pre-season precipitation (Cong et al., 2021). Water availability is important for herbaceous plant growth in arid and semi-arid regions, where water availability is often insufficient due to high soil salinity and low water content (Wang et al., 2022d) and where vegetation tends to become more sensitive to moisture conditions (Shen et al., 2015a, 2015, 2018). In addition, the presence of snow can serve to regulate the soil temperature at a considerably higher level than the air temperature (Freppaz et al., 2008; Groffman et al., 2001): the higher soil temperature as well as the sufficient water availability results in the advancement of the grassland SOS. As a result, the trend of advancement of grassland SOS gradually increased with the increasing trend of rising precipitation in winter while contributing to the increasing negative partial correlation between T_min_ and T_max_ and SOS in spring.

In contrast, in the warm, arid southwestern portion of Region 3 (mainly grasslands), the warm climatic conditions and the weak decreases in T_min_ during the winter do not serve to conserve the snowpack, and this, combined with the region’s reduced spring precipitation and poor moisture levels, and the lack of available water resulted in a delay in the emergence of grassland SOS in the region (Ren et al., 2017). In Region 4, encompassing the warm and humid Lesser Khingan Mountains and eastern Changbai Mountains, the decline in spring T_max_ made it difficult to meet the heat demand required for plant leaf unfolding, resulting in a delayed trend in DBF SOS; the severe heat deficit facilitated the spring temperature response of SOS when the decreasing trend in winter T_min_ in the region was reduced (Du et al., 2019b; Liu et al., 2024). In contrast, the response of SOS to spring temperatures in this region was promoted when the increase in soil temperatures due to winter snowpack led to an increase in the rising trend of winter precipitation. In addition, the southern part of Changbai Mountain is close to the ocean and has a warm and humid climate, and the increase in winter snow melts quickly in late winter, coupled with the increase in spring temperatures, which are good thermal and hydrological conditions that lead to the advancement of SOS in the region. Overall, the modulation of the efficiency of the spring temperature response of SOS by winter climate change and its modalities differed significantly between regions and vegetation types.

This study only considers the effects of temperature and precipitation as drivers of changes in vegetation phenology. Of course, geographic factors such as elevation can affect vegetation phenology (Shahzad et al., 2024). However, each of the main vegetation distribution areas in the study area falls within a limited elevation range, and any effects of elevation change are largely captured by changes in temperature (Ohmura, 2012). We also noted that socio-economic conditions and policy regulation should not be ignored (Marambe and Wijesundara, 2021), but given the relatively coarse (8 km × 8 km) pixel size used in our analysis, we cannot detect local variations that may be attributable to human activity (Gong et al., 2024; Piao et al., 2019). In future research, attempts should be made to combine remote sensing data with ground observations (Piao et al., 2020) and to integrate imagery from different types of sensors to explore the effects of geographic factors, as well as more climatic factors, on vegetation SOS.

## 5 Conclusions

This study demonstrates the relationships between climate indicators and changes in the start of the vegetation growing season in the high latitudes of Northeast China over recent decades.

First, we found that the SOS in high latitudes in China showed an overall trend of advancement, but the trend of SOS varied significantly in space and by vegetation types. The SOS in the cold north and the cold, arid northwestern–central parts of the study area advanced significantly with trends of 1.9 days/decade and 1.6 days/decade, respectively. These trends represent an increase in SOS advancement efficiency of 58% and 167% compared to the regional average SOS for DNF and grasslands, respectively. The SOS in the warm, arid southwest and warm, humid Lesser Khingan Mountains and Changbai Mountain regions in the eastern part of the study area were delayed with trends of 2.6 days/decade and 1.2 days/decade, respectively.

Second, warming had opposing effects on SOS depending on when it occurred. Decreasing spring temperatures constrain the initiation of vegetation SOS, contributing to the delay in DBF SOS in the Changbai Mountains. In contrast, decreases in winter temperature, mainly nighttime temperature, enhance the process of recovering from the dormancy phase, contributing to the higher efficiency of SOS response to spring temperatures and the asymmetric modulation of SOS spring temperature response efficiency by winter T_min_ and T_max_.

Third, the moderating effect of winter precipitation on the efficiency of the SOS spring temperature response differs with vegetation type. An increase in winter precipitation delayed the satisfaction of DNF cold demand in the cold northern region, which was detrimental to the breaking of vegetation dormancy, thus inhibiting the efficiency of the spring temperature response of the DNF SOS. For the cold, arid northwestern and central regions, an increase in snow cover improves the moisture conditions required for vegetation emergence and enhances the efficiency of the grassland SOS response to spring temperatures.

In summary, this paper demonstrates the moderating role of changes in winter climate in affecting the SOS responsiveness to spring temperature, and it emphasizes that the moderating role of winter climate should be considered when exploring vegetation phenology–spring climate connections. This knowledge can help to more precisely and reasonably predict temperature-driven phenological changes under future climate change.

## Data Availability

The original contributions presented in the study are included in the article/**Supplementary Material**. Further inquiries can be directed to the corresponding author.
