# Supplementary material for: Winter climate change mediates the sensitivity of vegetation leaf-out to spring warming in high latitudes in China
Source: Front Plant Sci. 2024 Dec 2;15:1476576. doi: 10.3389/fpls.2024.1476576 (PMC11646735; doi:10.3389/fpls.2024.1476576)
Supplement: Supplementary file 1 [file DataSheet1.pdf]

Supplementary Material

Supplementary Figures

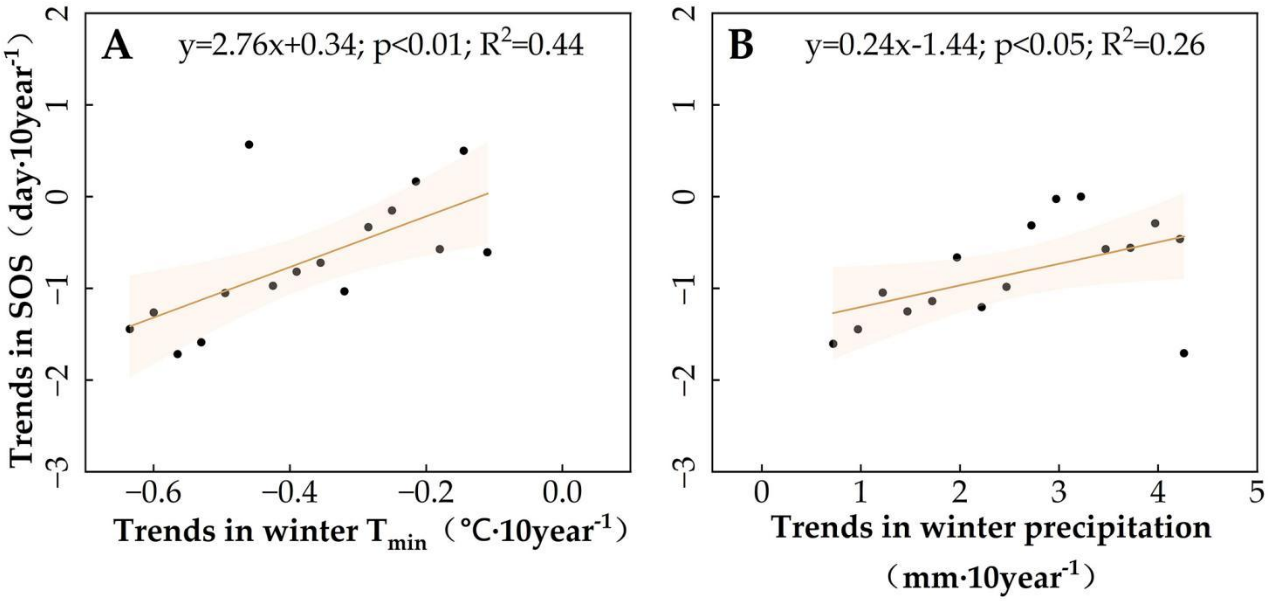

Figure S1. Moderating role of winter climatic indicators in Region 1 (DNF) on the spring temperature sensitivity of SOS; (A) the effect of winter T_{min} trends on SOS trends; (B) the effect of winter precipitation trends on SOS trends. The lines indicate the linear fit for the winter temperature trends, and the shading represents the 95% confidence band.

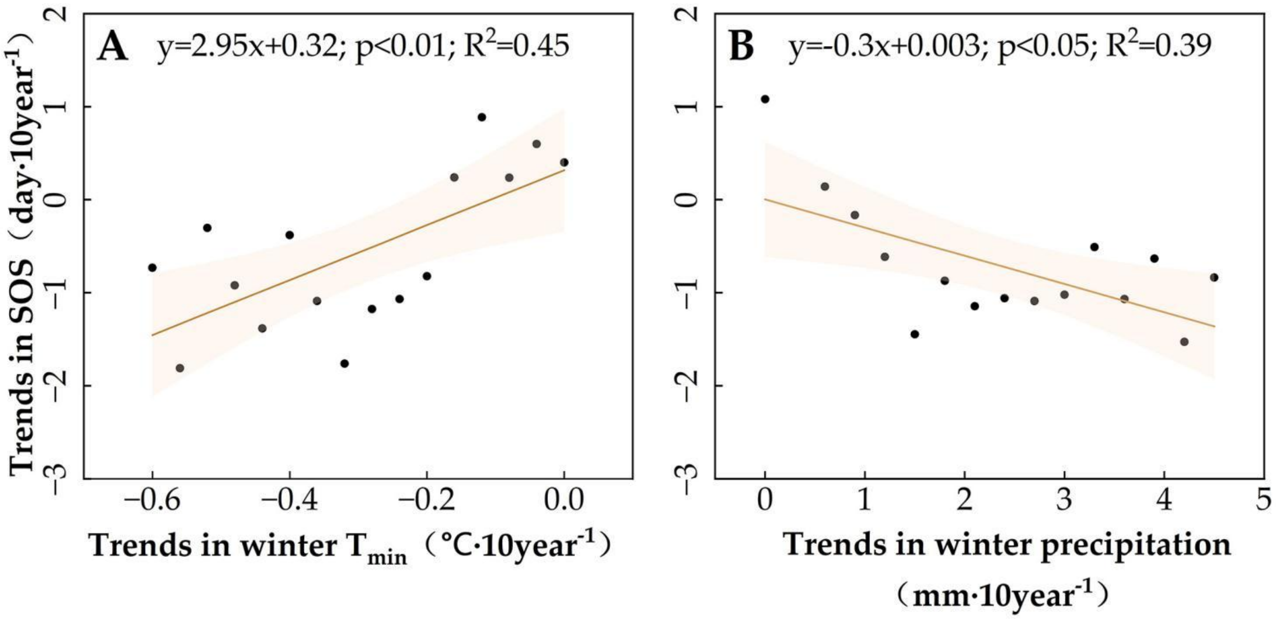

Figure S2. Moderating role of winter climatic indicators in Region 2 (grassland) on the spring temperature sensitivity of SOS; (A) the effect of winter T_{min} trends on SOS trends; (B) the effect of winter precipitation trends on SOS trends. The lines indicate the linear fit for the winter temperature trends, and the shading represents the 95% confidence band.

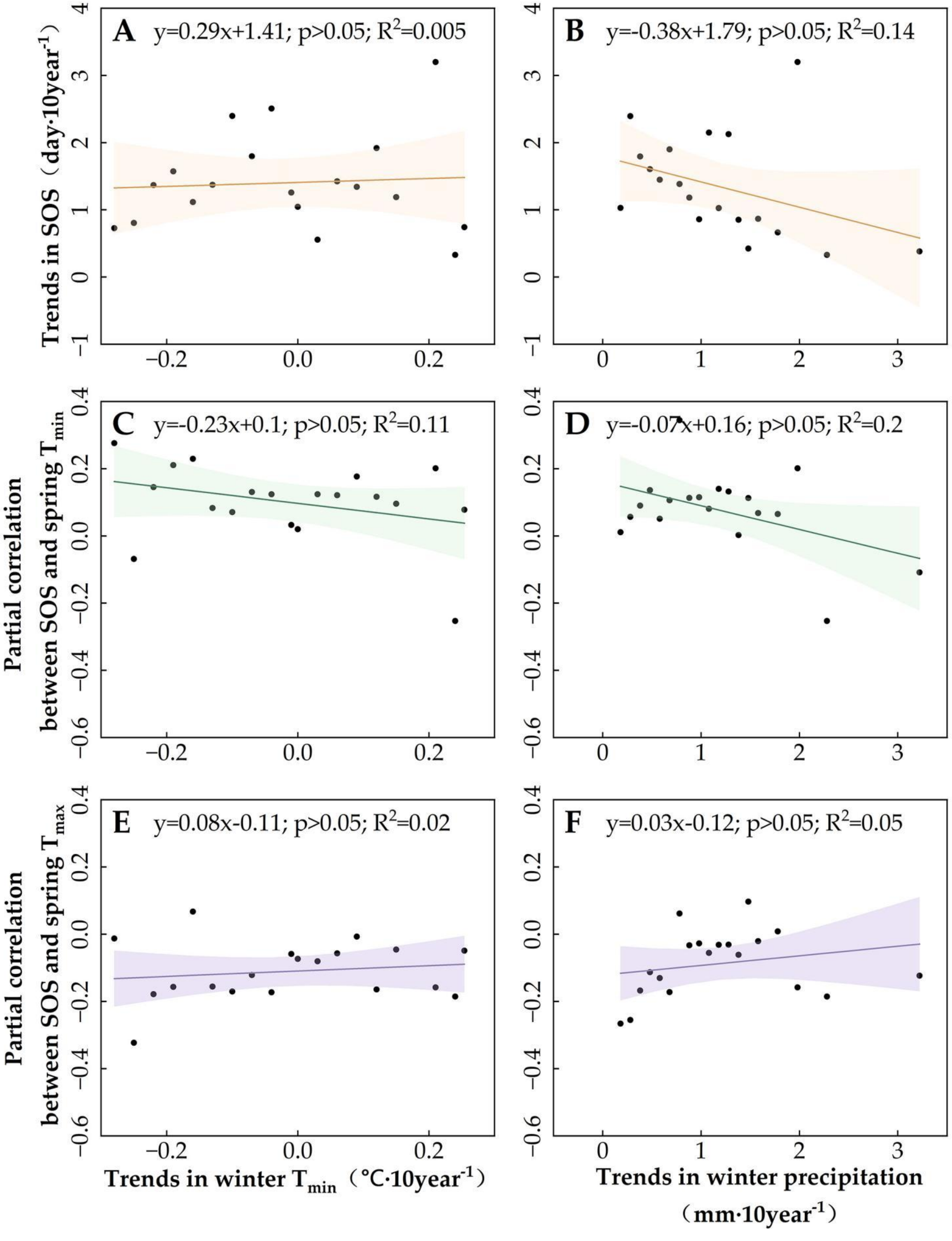

Figure S3. Moderating role of winter climatic indicators in Region 3 (grassland) on the spring temperature sensitivity of SOS; (A) the effect of winter T_{min} trends on SOS trends; (B) the effect of

winter precipitation trends on SOS trends; (C) the effect of winter T_{min} trends on the partial correlation between SOS and T_{min}; (D) the effect of winter precipitation trends on the partial correlation between SOS and T_{min}; (E) the effect of winter T_{min} trends on the partial correlation between SOS and T_{max}; (F) the effect of winter precipitation trends on the partial correlation between SOS and T_{max}. The lines indicate the linear fit for the winter temperature trends, and the shading represents the 95% confidence band.

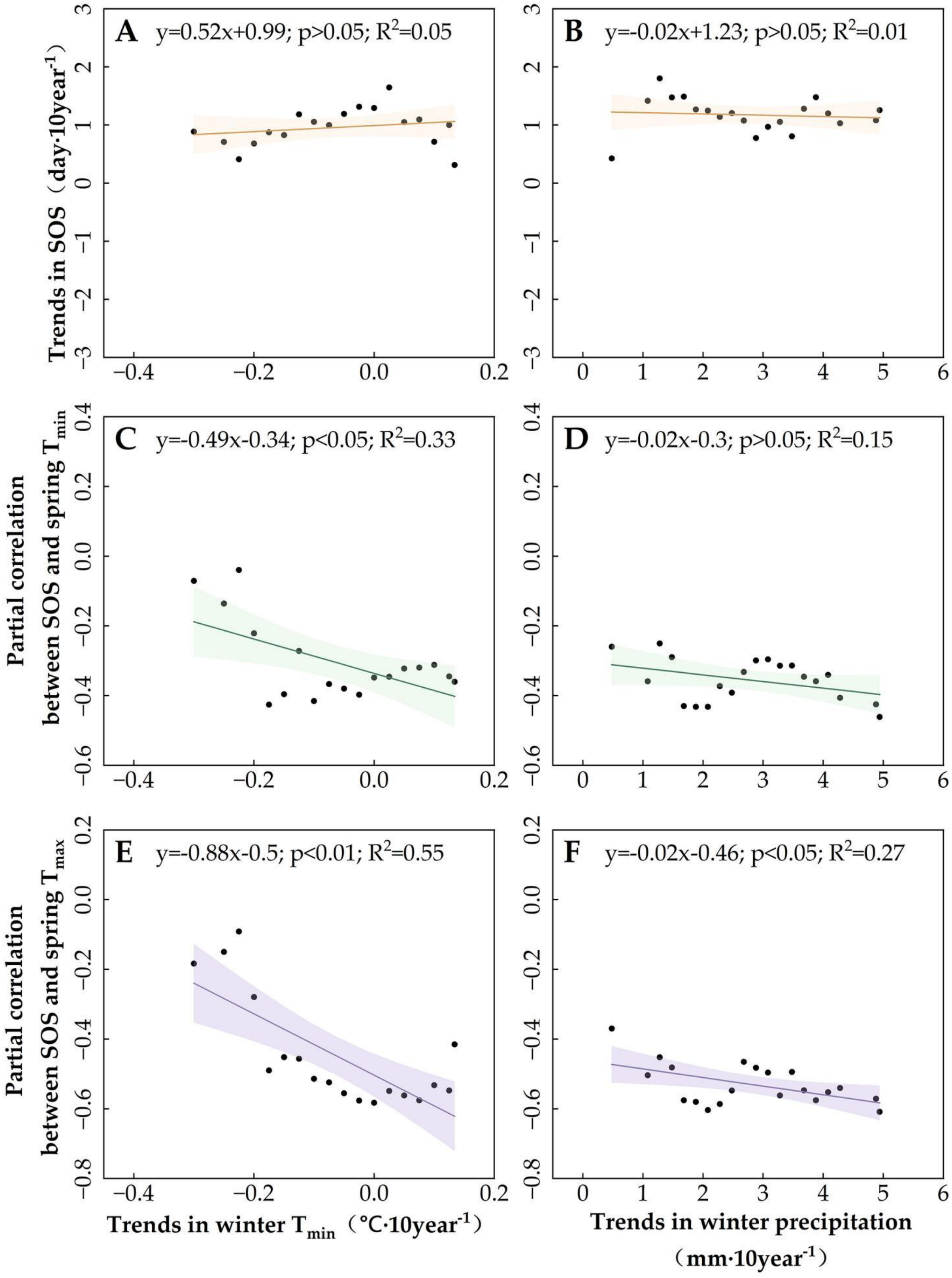

Figure S4. Moderating role of winter climatic indicators in Region 4 (DBF) on the spring

temperature sensitivity of SOS; (A) the effect of winter T_{min} trends on SOS trends; (B) the effect of winter precipitation trends on SOS trends; (C) the effect of winter T_{min} trends on the partial correlation between SOS and T_{min}; (D) the effect of winter precipitation trends on the partial correlation between SOS and T_{min}; (E) the effect of winter T_{min} trends on the partial correlation between SOS and T_{max}; (F) the effect of winter precipitation trends on the partial correlation between SOS and T_{max}. The lines indicate the linear fit for the winter temperature trends, and the shading represents the 95% confidence band.

Supplementary Tables

Table S1. Proportion of pixels with negative partial correlation between SOS and climate factors

		**DNF**	**DBF**	**Grassland**
T_{min}	Winter	45.12% (0.18%)	62.13% (0.3%)	52.97% (2.91%)
	Spring	99.42% (52.99%)	99.13% (65.24%)	63.39% (6.55%)
T_{max}	Winter	81.39% (0.49%)	91.83% (5.08%)	59.39% (4.36%)
	Spring	100% (85.26%)	99.85% (86.9%)	73.01% (15.8%)
Precipitation	Winter	96.27% (26.74%)	80.9% (18.55%)	88.28% (27.68%)
	Spring	85.66% (3.6%)	69.13% (39.78%)	84.28% (35.84%)

Note: Proportion of significant pixels in parentheses ($p<0.05$).

Table S2. Proportion of pixels with positive partial correlation between SOS and climate factors

		DNF	DBF	Grassland
T_{min}	Winter	54.88% (0.13%)	37.87% (0.17%)	47.03% (2.87%)
	Spring	0.58% (0)	0.87% (0)	36.61% (2.3%)
T_{max}	Winter	18.61% (0)	8.17% (0)	40.61% (1.01%)
	Spring	0	0.15% (0)	26.99% (0.4%)
Precipitation	Winter	3.73% (0.22%)	19.1% (1.35%)	11.72% (0.48%)
	Spring	14.34% (0)	30.87% (0.02%)	15.72% (0.24%)

Note: Proportion of significant pixels in parentheses ($p<0.05$).
